# Protective Effect of Qiliqiangxin against Doxorubicin-Induced Cardiomyopathy by Suppressing Excessive Autophagy and Apoptosis

**DOI:** 10.1155/2022/9926635

**Published:** 2022-01-27

**Authors:** Yating Qin, Chao Lv, Xinxin Zhang, Weibin Ruan, Xiangyu Xu, Chen Chen, Xiaoning Wan, Xinyun Ji, Juan Zhou, Li Lu, Xiaomei Guo

**Affiliations:** ^1^Department of Cardiology, Tongji Hospital, Tongji Medical College, Huazhong University of Science and Technology, Wuhan, 430030 Hubei, China; ^2^Department of Cardiology, The Second Hospital of Shandong University, Jinan, 250000 Shandong, China; ^3^Department of Cardiology, The Third People's Hospital of Hubei Province, Wuhan, 430030 Hubei, China; ^4^Department of Cardiology, Hubei Provincial Hospital of Traditional Chinese Medicine, Wuhan, 430073 Hubei, China; ^5^Department of Cardiology, Renmin Hospital of Wuhan University, Wuhan, 430030 Hubei, China

## Abstract

**Background:**

Doxorubicin (DOX) is one of the most potent and widely prescribed antitumor agents; however, its clinical use is limited by cardiac side effects. In this study, we aimed to clarify the protective effects of Qiliqiangxin (QL), a traditional Chinese medicine formulation, on DOX-induced cardiotoxicity and to explore the underlying mechanisms in a rat model.

**Methods:**

Male Sprague-Dawley rats were randomly assigned to three groups with different interventions (control, DOX, and DOX plus QL) for 31 days. Cardiac function was monitored. The levels of oxidative stress in plasm were detected, the activities of autophagy and apoptosis in rat hearts were determined, and then, the related PI3K/AKT/mTOR signal pathway regulating apoptosis and autophagy was investigated.

**Results:**

QL improved cardiac dysfunction and decreased the increased level of cardiac enzymes in plasm caused by DOX. Moreover, DOX exposure resulted in oxidative stress enhancement, which was suppressed by QL treatment. Then, we discovered that DOX intervention caused the apoptosis of cardiomyocytes by activating the mitochondrial-dependent apoptotic pathway which was strongly inhibited by QL treatment. Furthermore, there was a significant increase in autophagic activities in the DOX-stimulated myocardium. Administration of QL substantially inhibited the enhanced autophagic activities, which might be attributed to the activation of PI3K/AKT/mTOR cascade, followed by suppression of ULK1 activity.

**Conclusions:**

QL exhibited protective roles against DOX-induced cardiotoxicity possibly via mediating the PI3K/AKT/mTOR pathway, leading to inhibition of autophagy and subsequent apoptosis activities.

## 1. Introduction

Doxorubicin (DOX) is known as one of the most potent and widely prescribed chemotherapeutic drugs for the treatment of a wide spectrum of solid tumors and hematological malignancies [1]. However, the clinical application of DOX is limited by its dose-dependent adverse effects, in which cardiotoxicity is of particular concern [2, 3]. It has been documented that DOX-induced cardiotoxicity is ultimately manifested with irreversibly dilated cardiomyopathy and congestive heart failure [4]. Unfortunately, no clinically efficacious approaches are established to attenuate DOX-induced cardiotoxicity currently. Dexrazoxane, the only drug approved by the Food and Drug Administration (FDA) for the treatment of DOX-induced cardiotoxicity, has been removed from the market by the fact that it might cause the occurrence of secondary malignances [5, 6]. In view of this, new agents alleviating DOX-induced cardiotoxicity and improving cardiac dysfunction are urgently needed.

**Figure 1:**
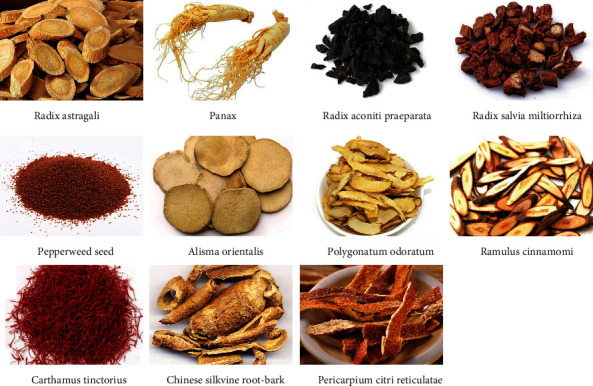
The morphology of 11 raw herbs that compose traditional Chinese medicine Qiliqiangxin capsule.

Qiliqiangxin (QL) capsule, a traditional Chinese medicine extracted from 11 distinct herbs including Radix Astragali, Aconite Root, Ginseng, Salvia miltiorrhiza, Semen Lepidii Apetali, Cortex Periplocae Sepii Radicis, Rhizoma Alismatis, Carthamus tinctorius, Polygonatum odorati, Seasoned Orange Peel, and Ramulus Cinnamomi, has been widely applied for the management of heart failure since it was approved by Chinese FDA in 2004 [7–9] (Figure 1). In spite of alleviating the development of heart failure, QL has been demonstrated to possess protective roles against a variety of cardiovascular diseases such as spontaneous hypertension [10], myocardial infarction [11], anoxic injury [12], and arrythmia [13], mainly through reducing cardiac hypertrophy and remolding, saving mitochondrial function, improving endothelial dysfunction, modulating iron channels, etc. However, whether QL plays beneficial effects on inhibiting DOX-induced cardiotoxicity is poorly understood.

Extensive studies have revealed that multiple potential pathological processes including excessive oxidative stress [14], mitochondrial dysfunction [15], myocardium apoptosis [16], and autophagy dysregulation [17] are involved in DOX-triggered cardiotoxicity; however, the mainly underlying molecular mechanisms are still controversial and remain to be fully elucidated. Therefore, this study was carried out to evaluate the potential therapeutic roles of QL against DOX-induced cardiotoxicity in rats and explore the underlying mechanisms.

## 2. Materials and Methods

### 2.1. Animal Procedure

**Figure 2:**
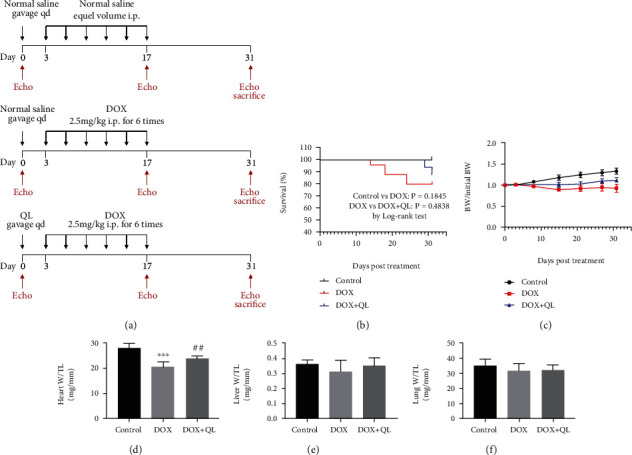
The impacts of QL treatment on survival rate, weight loss, cardiac mass, liver mass, and lung mass in DOX-treated rats. (a) The schematic diagram of this study. (b) Survival curve and (c) body weight change of rats in three groups during the experiment. (d–f) The ratios of heart weight, lung weight, and liver weight to tibial length (TL) among three groups at the end of the experiment (values are presented as mean ± SD; ^∗∗∗^*P* < 0.001 vs. control group; ^##^*P* < 0.001 vs. DOX group).

Adult 8-week-old male Sprague-Dawley rats, weighing 200-220 g, were purchased from the Laboratory Animal Center of Huazhong University of Science and Technology, Wuhan, China. All rats were housed in specific pathogen-free (SPF) facilities under a controlled temperature (22 ± 2°C) and humidity with a standard diet and tap water *ad libitum*. Animals were randomly separated into 3 groups after acclimation for one week and then were treated as follows: rats in the control group (*n* = 15) were given equal volume of saline by intraperitoneal injection or gavage; rats in the DOX group (*n* = 15) were intraperitoneally injected with a total of 15 mg/kg doxorubicin (Shenzhen Main Luck Pharmaceuticals Inc., China) within 2 weeks (2.5 mg/kg per time for 6 times) and orally administered equal volume of saline; rats in the DOX+QL group (*n* = 15) were given 1 g/kg QL (Shijiazhuang Yiling Pharmaceuticals Inc., China) dissolved in saline via gavage per day beginning 3 days before the initiation of DOX injection for 31 days. On the 31st day, all animals were subjected to echocardiography evaluation, followed by anesthetization, and sacrificed by intraperitoneal injection of 100 mg/kg pentobarbital sodium (Figure 2(a)).

Blood samples were collected, and the hearts were rapidly removed after rats were anesthetized. Blood samples were centrifuged at 4°C for 10 min at 3000 rpm to obtain the serum samples, which then were immediately stored at -80°C until biochemical analysis. Being cut into several pieces, some of the excised hearts were immersed in 10% formaldehyde for subsequent histological analysis, some were immersed in 2.5% glutaraldehyde for electron microscopy analysis, and the others were frozen in liquid nitrogen and stored at -80°C until use. All animal experimental protocols were approved by the Institutional Animal Care and Use Committee of Tongji Medical College, Huazhong University of Science and Technology, Wuhan, China (the IACUC Number is 2559), and strictly adhered to the National Institutes of Health Guide for the Care and Use of Laboratory animals.

### 2.2. Echocardiography Evaluation

Transthoracic echocardiography (VisualSonics, Canada) was conducted for left ventricular function evaluation on the 0th, 17th, and 31st days in all animals. Rats were anesthetized with pentobarbital sodium and tied on a heated platform, with their chest hairs removed. M-mode images were recorded, and echocardiographic parameters including left ventricular end-systolic diameter (LVEDs), left ventricular end-diastolic diameter (LVEDd), left ventricular posterior wall thickness in end-diastole (LVPWd), left ventricular posterior wall thickness in end-systole (LVPWs), left ventricular anterior wall thickness in end-diastole (LVAWd), and left ventricular anterior wall thickness in end-systole (LVAWs) were determined. Left ventricular ejection fraction (EF) and left ventricular fractional shortening (FS) were calculated as follows: EF = (LVEDd^3^ − LVEDs^3^/LVEDd^3^) × 100% and FS = (LVEDd − LVEDs)/LVEDd × 100%.

### 2.3. Histopathological Analysis

Hearts were immediately excised, weighed, washed in cold phosphate-buffered saline, and cut into several pieces. The left ventricular sections were fixed in 4% paraformaldehyde at room temperature for 24 h. Then, the samples were dehydrated and embedded in paraffin blocks, and cross-sections at 5 *μ*m thick were obtained. For evaluation of heart pathological morphology and myocardial fibrosis, hematoxylin and eosin (H&E) staining and Masson's trichrome staining were conducted following the manufacturer's instructions (Servicebio, Wuhan, China). For evaluation of cardiomyocyte size, paraffin-embedded sections were deparaffinized, rehydrated in xylene and graded ethanol, and then subjected to wheat germ agglutinin (WGA) staining according to the manufacturer's protocol (Servicebio, Wuhan, China). All images were acquired using an optical microscope (Olympus, Tokyo, Japan) and analyzed by Image-Pro Plus 6.0 software.

### 2.4. Immunohistochemical and Immunofluorescent Analysis

For immunohistochemistry or immunofluorescent staining, paraffin-embedded heart sections were deparaffinized, rehydrated in xylene and graded ethanol, treated with antigen retrieval solution in boiled water for 20 min, and subjected to hydrogen peroxide for 30 min after cooling down. Tissue sections were blocked with 5% BSA in room temperature for 1 h prior to incubation overnight at 4°C with primary antibodies against cleaved caspase-3 (Cell Signaling Technology 9661) and LC3 BII (Cell Signaling Technology 12741). After incubation with secondary antibody, for immunohistochemical analysis, tissue sections were stained with diaminobenzidine (DAB) (Servicebio, Wuhan, China) and counterstained with hematoxylin, followed by light microscope observation (Olympus); for immunofluorescent analysis, tissue section images were captured by a fluorescent microscope (Olympus). The positive staining areas were calculated and analyzed by Image-Pro Plus 6.0 software.

### 2.5. Terminal Deoxynucleotidyl Transferase-Mediated dUTP Nick End Labeling (TUNEL) Staining

Paraffin-embedded heart sections were stained with the One Step TUNEL Apoptosis Assay Kit (Dalian Meilun Biotechnology, China) to evaluate the apoptosis of myocardial cells stimulated by DOX. The average apoptotic index was defined as the ratio of the number of TUNEL-positive myocytes relative to DAPI-stained myocytes from 6 random fields in the section.

### 2.6. Transmission Electron Microscopy Analysis

Left ventricular sections in 1 mm^3^ were quickly cut and immersed in 2.5% glutaraldehyde with 0.1 M sodium cacodylate buffer (Servicebio, Wuhan, China) at room temperature for 2 h and then stored overnight at 4°C. The sections were washed 3 times in 0.1 M sodium cacodylate buffer and postfixed in 1% osmium tetroxide with 0.1 M sodium cacodylate buffer (Servicebio, Wuhan, China). After being dehydrated in ethanol and embedded in resin, ultrathin sections were counterstained with uranyl acetate and imaged on a transmission electron microscope (Hitachi-HT7800, Japan).

### 2.7. Biochemical Index Analysis

Serum levels of creatine kinase isoenzyme-MB (CK-MB) and cardiac troponin T (cTnT) were detected using ELISA kits (mlbio, Shanghai, China) in accordance with the manufacturer's instructions. The measurements of serum levels of aspartate aminotransferase (AST), alanine aminotransferase (ALT), lactate dehydrogenase (LDH), serum superoxide dismutase (SOD), nitric oxide (NO), total antioxidant capacity (T-AOC), reduced glutathione (GSH) activities, and hydrogen peroxide (H_2_O_2_) were performed using commercially available assay kits (Nanjing Jiancheng Bioengineering Institute, Nanjing, China) according to the manufacturer's protocols.

### 2.8. Western Blot

For extraction of the total protein, left ventricular heart tissues from rats were homogenized in commercial RIPA buffer (Boster, Wuhan, China) added with protease inhibitor and phosphatase inhibitor (MedChemExpress, America). Then, the supernatants were obtained after centrifugation at 12000×*g* at 4°C for 20 min. The protein concentration was detected using a bicinchoninic acid assay (Boster, Wuhan, China). Denatured proteins (40 *μ*g/lane) were loaded on and separated by 8-10% sodium dodecyl sulfate-polyacrylamide electrophoresis gels and transferred onto polyvinylidene difluoride membranes. Then, the membranes were blocked with 5% BSA at room temperature for 1 h, which subsequently were incubated with primary antibodies overnight at 4°C. After washing with TBS-T, the membranes were probed with peroxidase-conjugated secondary antibodies (Boster, Wuhan, China) at room temperature for 1 h. The protein bands were visualized using an enhanced chemiluminescence (ECL) kit following the manufacturer's instructions (New Cell & Molecular Biotech, Suzhou, China). Protein expression contents were analyzed by ImageJ software, and the level of *β*-actin or GAPDH was used as an internal control. The primary and secondary antibodies used in this study were listed in Supplementary Table 1.

### 2.9. Statistical Analysis

The data in this study was presented as mean ± standard deviation (SD). The differences among the groups were assessed using a one-way ANOVA test. All of the data analysis was performed with GraphPad Prism software version 8.0. A *P* value less than 0.05 was considered statistically significant.

## 3. Results

### 3.1. QL Ameliorated DOX-Induced Body Weight and Cardiac Mass Loss in Rats

To explore the effects of QL on regulating DOX-induced cardiac damage, the rats treated with DOX intraperitoneal injection were used as the in vivo animal model of cardiotoxicity. Firstly, we analyzed the survival rate of rats in different groups and found that there were no significant differences among the three groups (Figure 2(b)). Unlike the highly lethal acute cardiac dysfunction, we established a subclinical cardiac dysfunction model of rats that mimicked an observed clinically chronic DOX-related cardiotoxicity, which might be responsible for the undifferentiated mortality of rats in this study, just as the results reported previously [18]. Then, in accordance with clinical evidence of body weight loss in DOX-treated patients [19, 20], DOX resulted in a 20% reduction of body weight in rats, while this pathological phenomenon was effectively improved by administration of QL (Figure 2(c)). Moreover, as shown in Figures 2(d)–2(f), DOX treatment dramatically decreased the ratio of heart weight to tibial length (heart W/TL) when compared with the control group, yet QL intervention significantly increased heart W/TL up to the level of that in the control group when compared with the DOX group. These findings, along with the fact that DOX triggered cardiomyocyte apoptosis and myocardium atrophy [21], accompanied by heart chamber enlargement and mass reduction [22], suggested that QL probably inhibited DOX-induced myocardial death. However, there were no significant differences in the ratios of lung weight to tibial length (lung W/TL) and the ratio of liver weight to tibial length (liver W/TL) among three groups, which indicated that there were no lung and liver congestion in these rats.

### 3.2. QL Attenuated Left Ventricular Dysfunction and Cardiac Injury in DOX-Treated Rats

**Figure 3:**
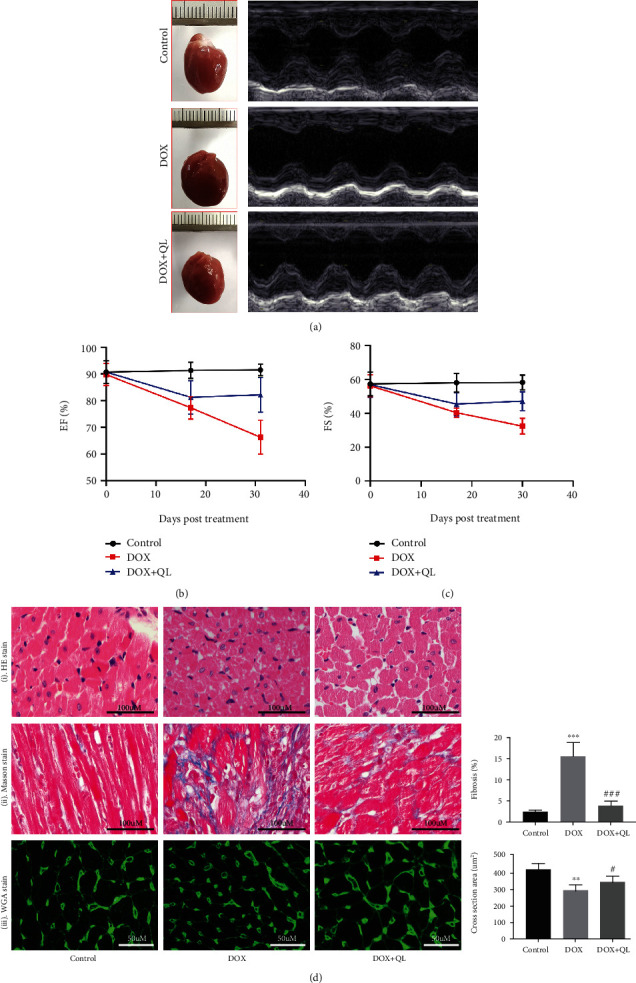
QL improved cardiac dysfunction and histopathological changes in rats under DOX exposure for 28 days. (a) Representative M-mode images of rats in different groups at the end of the experiment. (b, c) Left ventricular ejection fraction (EF%) and left ventricular fractional shorting (FS%) of rats in three groups on the 0th, 17th, and 31st day of the trial. (d) Histological analysis of heart tissues in three groups. (i) Representative HE staining displaying transverse myocardial section (300 dpi, scale bar: 100 *μ*M). (ii) Masson staining of heart tissues. Collagen content was shown as fibrosis (300 dpi, scale bar: 100 *μ*M). (iii) WGA staining of heart tissues (300 dpi, scale bar: 50 *μ*M). Cardiomyocyte sizes were represented by cross-section areas (values are presented as mean ± SD; ^∗∗^*P* < 0.01 and ^∗∗∗^*P* < 0.001 vs. control group; ^#^*P* < 0.05 and ^##^*P* < 0.001 vs. DOX group; *n* = 5 per group).

**Figure 4:**
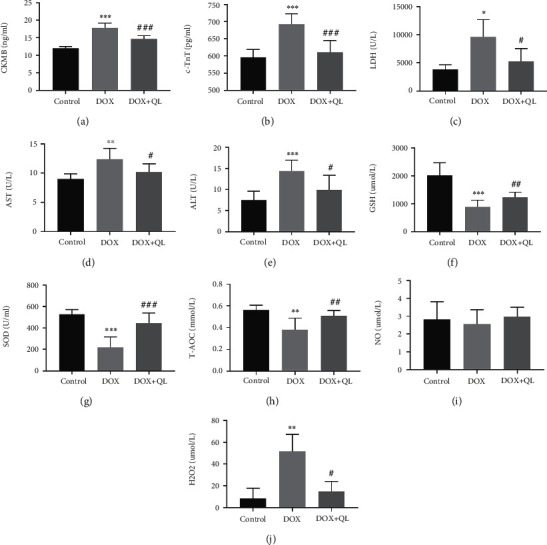
QL attenuated cardiac injury and oxidative stress in circulation in DOX-treated rats after DOX treatment for 28 days. (a–e) The concentrations of cardiac injury parameters in plasm. (f–j) The concentrations of oxidative stress-related molecules in plasm in three different groups (values are presented as mean ± SD; ^∗^*P* < 0.05, ^∗∗^*P* < 0.01, and ^∗∗∗^*P* < 0.001 vs. control group; ^#^*P* < 0.05, ^##^*P* < 0.01, and ^###^*P* < 0.001 vs. DOX group; *n* = 10 per group). CKMB: creatine kinase isoenzyme-MB; c-TnT: cardiac troponin T; LDH: lactate dehydrogenase; AST: aspartate aminotransferase; ALT: alanine aminotransferase; GSH: reduced glutathione; SOD: serum superoxide dismutase; T-AOC: total antioxidant capacity; NO: nitric oxide; H_2_O_2_: hydrogen peroxide.

Left ventricular function parameters of rats in three groups on the 31st day were assessed by echocardiography (Figure 3(a)). As shown in Table 1, DOX exposure resulted in impaired left ventricular systolic function, as shown by increased LVEDs, and decreased EF%, FS%, LVPWs, and LVAWs in the DOX group when compared with that in the control group, whereas QL treatment markedly improved left ventricular systolic dysfunction induced by DOX. Interestingly, the left ventricular diastolic function of rats in three groups showed no statistical difference. Furthermore, we analyzed the levels of EF% and FS% at different periods of experiment and discovered that QL significantly preserved the progressively decreased levels of EF% and FS% induced by DOX, as depicted in Figures 3(b) and 3(c). In addition, histopathological examinations including HE staining, Masson staining, and WGA staining uncovered that DOX treatment significantly increased myocardial atrophy and tissue fibrosis and reduced cardiomyocyte size, which were improved by administration of QL (Figure 3(d)). Moreover, the levels of cardiac injury biomarkers like CK-MB, c-TnT, LDH, AST, and ALT were elevated in the circulation in the DOX-treated group when compared with that in the control group, while QL treatment effectively reduced the increased levels of above biomarkers (Figures 4(a)–4(e)).

### 3.3. QL Suppressed DOX-Induced Oxidative Stress in Rats

Considering that oxidative stress displayed an important role in facilitating DOX-triggered cardiac damage [23], concentrations of endogenous antioxidants and oxidative factors including SOD, GSH, T-AOC, NO, and H_2_O_2_ in the blood were detected. We discovered that DOX treatment caused significant decreases in serum levels of SOD, GSH, and T-AOC when compared with the control group, while administration of QL normalized these decreases (Figures 4(f)–4(j)). However, although DOX tended to reduce the level of circulating NO and QL treatment tended to reverse the effects of DOX, there were no significantly statistical differences in NO content among the three groups. Moreover, rats in the DOX group showed a higher concentration of H_2_O_2_ in serum than that in the QL intervention group and the control group. The findings above at least partly declared that QL was capable of attenuating oxidative stress in DOX-treated rats in some extent, which might be partly responsible for the cardioprotective actions of QL.

### 3.4. QL Inhibited DOX-Induced Apoptosis in Rat Hearts

**Figure 5:**
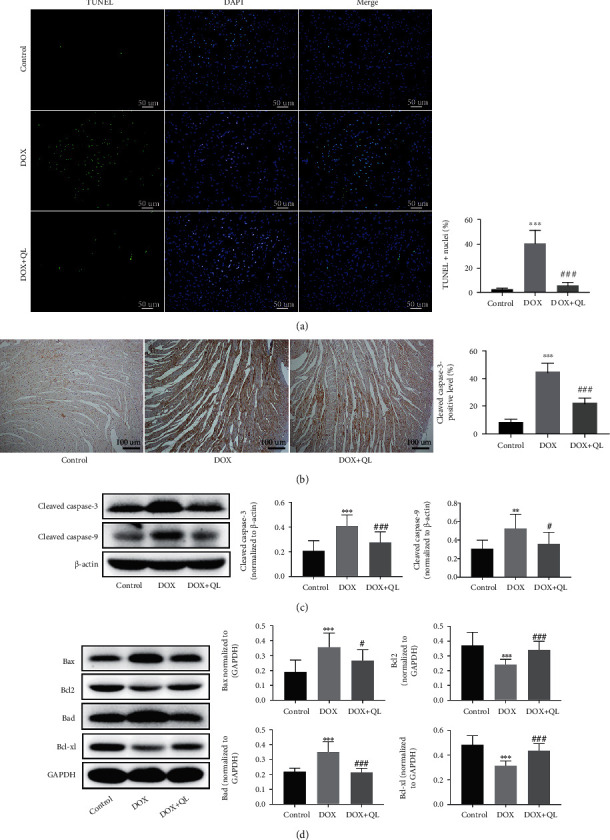
QL inhibited DOX-induced cardiomyocyte apoptosis in rats under DOX exposure for 28 days. (a) TUNEL staining of cardiomyocytes in three groups. Positive nuclei were stained in green, and nuclei were stained in blue (*n* = 7 per group, 300 dpi, scale bar: 50*μ*m). (b) Representative immunohistochemical images of cleaved caspase-3 expression in heart tissues in three groups (*n* = 4 per group, 300 dpi, scale bar: 100*μ*m). (c, d) The expression levels of cleaved caspase-3, cleaved caspase-9, Bax, Bcl-2, Bcl-xl, and Bad in different group heart tissues by western blot (*n* = 6) (values are presented as mean ± SD; ^∗∗^*P* < 0.01 and ^∗∗∗^*P* < 0.001 vs. control group; ^#^*P* < 0.05, ^##^*P* < 0.01, and ^###^*P* < 0.001 vs. DOX group).

We further explored the potential mechanisms of QL against DOX-induced myocardial injury. Owing to that cardiac apoptosis was one of the established pathological hallmarks in DOX-induced cardiotoxicity [21], we investigated the role of QL in DOX-induced myocardial apoptosis in rat hearts. As shown in Figure 5(a), TUNEL staining indicated that the percentage of apoptotic cells in DOX-treated myocardium tissue was significantly increased, while QL treatment dramatically suppressed the increased apoptosis. Immunohistochemical analysis of heart tissue suggested that the expression of cleaved caspase-3, a vital proapoptotic factor, was obviously increased in the DOX-treated group when compared with the control group, while QL intervention efficaciously suppressed the increase (Figure 5(b)). Similar findings were observed in western blot analysis of heart tissues; the expressions of proapoptotic proteins including cleaved caspase-3, cleaved caspase-9, Bax, and Bad were found to be much lower in the coadministration group than that in the DOX alone treatment group. On the contrary, antiapoptotic factors including Bcl-2 and Bcl-xl were expressed much higher in the hearts of the coadministration group than that in the DOX alone treatment group (Figures 5(c) and 5(d)). The results above strongly suggested that QL could attenuate DOX-induced myocardial apoptosis.

### 3.5. QL Alleviated DOX-Induced Excessive Autophagy in Rat Hearts

**Figure 6:**
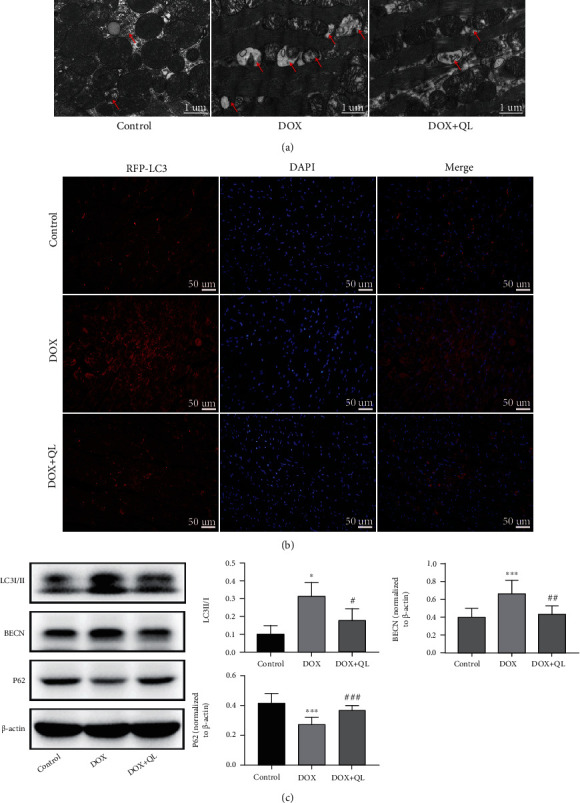
QL reduced DOX-induced excessive autophagy in rat hearts. (a) Representative electron microscopy images of heart tissues in different groups. The red arrows refer to autophagic vacuoles containing damaged organelles (300 dpi, scale bar: 1 *μ*m). (b) Immunofluorescence images of LC3 II expression in different group heart tissues (300 dpi, scale bar: 50 *μ*m). (c) The expression levels of autophagy-related molecules including LC3 I/II, BECN, and P62 in different group heart tissues by western blot (*n* = 6) (values are presented as mean ± SD; ^∗^*P* < 0.05 and ^∗∗∗^*P* < 0.001 vs. control group; ^#^*P* < 0.05, ^##^*P* < 0.01, and ^###^*P* < 0.001 vs. DOX group).

It was demonstrated that dysfunction of autophagy in the myocardium contributed to the development of several cardiovascular diseases including DOX-induced cardiotoxicity via favoring the apoptosis of myocardial cells [24, 25]. Then, we detected the effects of QL on autophagic activities in DOX-treated rat hearts. The results of electron microscopy showed that there were highly accumulated autophagic vacuoles containing damaged organelles in the heart tissue of rats under DOX exposure when compared with the control group, and these structures were much less abundant post-QL treatment (Figure 6(a)). Immunofluorescence analysis revealed that the level of LC3 II, a hallmark of autophagosome formation, was dramatically increased in DOX-treated rat hearts; conversely, QL intervention significantly inhibited LC3 II accumulation (Figure 6(b)). In line with the findings above, western blot analysis of heart tissue indicated that the expressions of autophagic molecules including LC3 II and BECN were obviously increased in the DOX group compared with the control group, while the administration of QL had a markedly suppressive effect on the expressions of these molecules. In contrast, the expression of P62 was observed much lower in the DOX group than that in the coadministration group (Figure 6(c)). These results demonstrated the effective roles of QL in weakening the enhanced autophagic activities in DOX-exposed heart tissue, indicating that QL possibly suppressed cardiomyocyte apoptosis through restraining the progression of excessive autophagy, thereby ameliorating myocardial damage induced by DOX.

### 3.6. QL Activated Cardiac PI3K/AKT/mTOR Signaling Pathway in DOX-Treated Rats

**Figure 7:**
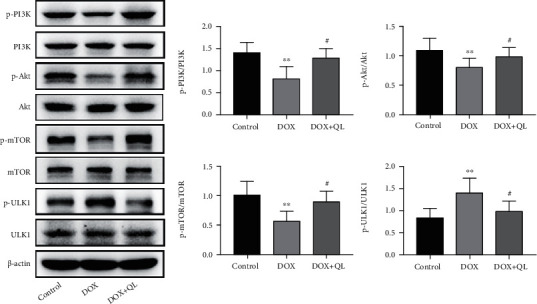
QL activated cardiac PI3K/AKT/mTOR signaling pathway in DOX-treated rats. The expression levels of p-PI3K, PI3K, p-Akt, Akt, p-mTOR, mTOR, p-ULK1, and ULK1 in different group heart tissues by western blot (*n* = 6) (values are presented as mean ± SD, ^∗∗^*P* < 0.01 vs. control group; ^#^*P* < 0.05 vs. DOX group).

Then, to illustrate potential mechanisms involving in protective effects of QL against DOX-induced cardiac damage, the upstream molecules regulating autophagic activities were explored. As growing evidence indicated that mTOR played a pivotal role in mediating the initiation of autophagic activities, and PI3K/AKT signal axis was responsible for regulating the activation of mTOR [24, 26, 27], we investigated the signaling transduction of PI3K/AKT/mTOR cascade in DOX-treated rat hearts (Figure 7). In comparison with the control group, DOX markedly decreased the level of phosphorylated mTOR in rat hearts, yet QL treatment effectively restored the expression of phosphorylated mTOR. Similarly, DOX dramatically reduced the phosphorylation levels of PI3K and AKT in the myocardium compared with the control group, while QL treatment significantly enhanced the phosphorylated levels of PI3K and AKT compared with the DOX group. Moreover, it was demonstrated that signal transduction of the PI3K/AKT/mTOR pathway displayed negative roles in regulating the activity of ULK1 [24], a key factor involved in autophagy development. As expected, ULK1 was found to be highly expressed in DOX-treated heart tissues compared with the control group, while QL intervention significantly reduced the increased expression of ULK1 stimulated by DOX. The above findings suggested that QL suppressed the enhanced autophagy in DOX-treated rat hearts at least partly through mediating the PI3K/AKT/mTOR signaling pathway.

## 4. Discussion

**Figure 8:**
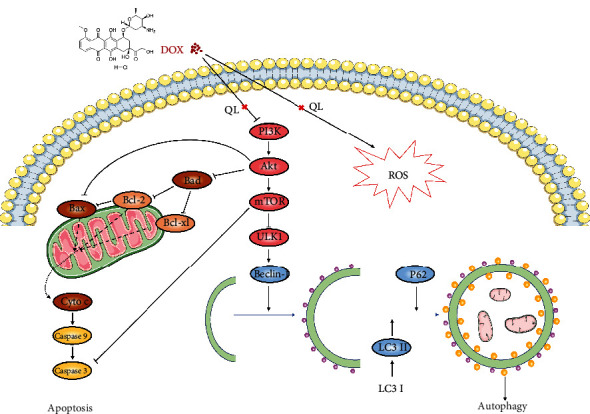
The schematic illustration of the potential molecular mechanisms by which QL alleviated DOX-induced cardiotoxicity.

QL, known as a traditional Chinese medicine formulation, has been widely used in Asian areas for the management of cardiovascular diseases for decades. A majority of studies have demonstrated the cardioprotective roles of QL in adverse cardiac remodeling, hypertrophy, or hypoxia-triggered injury; however, whether QL plays therapeutic roles in DOX-induced cardiotoxicity is poorly understood so far. In this study, DOX exposure resulted in cardiac damage and dysfunction in rats manifesting as increased myocardium atrophy and fibrosis, increased levels of cardiac injury enzymes, and decreased left ventricular systolic function, whereas QL intervention effectively improved these adverse effects induced by DOX. Then, we found that the protective roles of QL against DOX-induced cardiotoxicity were ascribed to suppressing myocardial excessive autophagy and apoptosis, in which the PI3K/AKT/mTOR signaling pathway is involved (Figure 8).

Oxidative stress was once the most widely accepted hypothesis that initiated DOX-induced cardiotoxicity [28]. DOX was demonstrated to induce excessive generation of reactive oxygen species (ROS) via electron exchange with oxygen molecules and redox cycling in the heart, thus resulting in oxidative stress and subsequently cardiomyocyte damage [15]. In this study, we observed that DOX administration triggered the redox imbalance in rats, as shown by reduced levels of antioxidant enzymes including SOD, GSH, and T-AOC, and increased level of H_2_O_2_ in the circulation. Then, QL treatment strongly attenuated DOX-induced oxidative injury evidenced by normalizing blood contents of SOD, GSH, T-AOC, and H_2_O_2_. However, previous studies demonstrated that neither antioxidants nor iron chelation could prevent DOX-induced cardiomyopathy [29, 30], strongly suggesting that oxidative stress was just one of the contributors in DOX-induced cardiotoxicity and QL could suppress DOX-activated oxidative stress.

Apoptosis is one type of highly programmed process of cell death, which is a normal physiologic process of cells; however, excessive apoptosis can be greatly harmful. Enhanced myocardial apoptosis was observed and demonstrated in several cardiovascular diseases, including myocardial ischemia/reperfusion injury, myocardial infarction, and DOX-induced cardiotoxicity, and inhibition of caspase activities could limit cardiac injury cardiomyocytes [31–34]. The mitochondrial-dependent apoptotic pathway, one of the three classic apoptotic signaling pathways, was reported involving pathogenesis of DOX-induced cardiotoxicity [35]. DOX exposure resulted in the opening of mitochondrial permeability transition pore, followed by the release of cytochrome C from mitochondria to cytosol. Then, cytochrome C binds to Apaf-1 and activates caspase-9, which successively activates caspase-3, thus initiating and amplifying apoptotic processes [36]. Moreover, proteins of the BCL-2 family regulated the development of mitochondrial-related apoptosis, among which proapoptotic molecules like Bax and Bad increase the permeability of mitochondrial membrane, while antiapoptotic molecules like Bcl-2 and Bcl-xl possess the reverse functions [36]. In line with previous studies, our results showed that DOX insult led to enhanced myocardial apoptosis, as evidenced by increased number of TUNEL-positive stained cells; increased expressions of cleaved caspase-9, cleaved caspase-3, Bax, and Bad; and decreased expressions of Bcl-2 and Bcl-xl in DOX-treated myocardial tissue in rats. However, QL treatment obviously normalized the aberrant expressions of molecules above, strongly suggesting that QL could attenuate DOX-induced cardiotoxicity by suppressing enhanced myocardial apoptosis through a mitochondrial-dependent apoptotic pathway.

Autophagy is an intracellular conserved mechanism by which the damaged organelles and misfolded proteins are degraded and recycled in order to maintain homeostasis and normal functions of cells [37]. Autophagy has been widely explored in diverse pathologies, such as neurodegenerative diseases, cancer, muscle diseases, aging, and cardiac diseases [38]. Several autophagic markers were elucidated after decades of investigation. LC3 II, the lipidation form of LC3 I, incorporates into autophagosome and facilitates its maturation, making itself a crucial marker for autophagy detection [39]. BECN is one of the essential regulators for autophagosome formation [27]. In addition, P62, which interacts with LC3 II for transporting marked cargos into autophagosome for degradation, is reported to be degraded by autolysosome, thus making itself another pivotal biomarker of autophagy [40, 41]. Whether autophagy is cytoprotective or cytotoxic remains controversial, which differs from different type of cells and severity extent of diseases [38]. For example, it was reported that BECN-dependent autophagy protected the heart from sepsis [42], while other researchers observed excessive autophagy in myocardial ischemia/reperfusion injury development [43]. Autophagy could be immediately activated by stresses like oxidative stress, and excessive autophagy could trigger the occurrence of cellular apoptosis [44, 45]. Here, we discovered that autophagic activities were remarkably enhanced in DOX-treated rat hearts, as evidenced by increased autophagic vacuole formation in cardiac tissues, accompanied with increased expression levels of LC3 II and BECN and decreased expression level of P62. Then, QL treatment induced the decrement of autophagic vacuole formation and reversed the abnormal expression of molecules involved in autophagic activities. Furthermore, growing evidence clarified that the autophagic activities were regulated by certain signaling axes, like TGFB-INHB/activin signaling [46], ERK-MAPK signaling [47], and PI3K/AKT/mTOR signaling [48]. However, the PI3K/AKT-related mTOR pathway is of particular importance. It has been illustrated that several bioactive factors including cytokines and ROS could inactivate PI3K/AKT/mTOR cascade, followed by the increased activity of downstream ULK1, a vital functional protein responsible for facilitating autophagy development [48, 49]. Here, we found that DOX administration led to the decreased expression of phosphorylated PI3K, AKT, and mTOR and the increased expression of phosphorylated ULK1. Yet, QL treatment enhanced the activities of PI3K, AKT, and mTOR signaling and inhibited the activation of ULK1. Taken together, these findings uncovered that QL exerted cardioprotective effects probably via improving DOX-evoked pathogenic events like oxidative stress and then amplifying the signal transduction of the PI3K/AKT/mTOR pathway, thereby suppressing the enhanced autophagic activities and subsequently encumbering the initiation of mitochondrial-related apoptotic cascade.

## 5. Conclusion

In conclusion, the cardioprotective effects of QL against DOX-induced cardiotoxicity in rats were at least partly ascribed to the regulation of PI3K/AKT/mTOR signaling cascade, leading to inhibition of autophagy and downstream apoptosis activities. These findings provide the evidence that QL has potentials to serve as a promising candidate for the treatment of myocardial damage triggered by DOX.

## Figures and Tables

**Table 1:** Left ventricular functional parameters of rats on 31st day.

	Control	DOX	DOX+QL
LVEDd (mm)	6.11 ± 0.23	6.53 ± 0.40	6.49 ± 0.22
LVEDs (mm)	2.55 ± 0.11	4.42 ± 0.29^∗∗∗^	3.61 ± 0.26^#^
LVPWd (mm)	1.67 ± 0.10	1.67 ± 0.07	1.85 ± 0.12
LVPWs (mm)	3.09 ± 0.20	2.25 ± 0.12^∗^	3.02 ± 0.16^#^
LVAWd (mm)	1.84 ± 0.09	1.58 ± 0.09	1.71 ± 0.11
LVAWs (mm)	3.19 ± 0.11	2.40 ± 0.15^∗∗∗^	2.67 ± 0.16
FS (%)	58.20 ± 1.38	32.43 ± 1.77^∗∗∗^	44.71 ± 2.92^##^
EF (%)	91.50 ± 0.69	66.29 ± 2.39^∗∗∗^	80.29 ± 2.93^###^

Data are presented as mean ± SD. DOX vs. control ^∗^*P* < 0.05 and ^∗∗∗^*P* < 0.001. DOX+QL vs. DOX ^#^*P* < 0.05, ^##^*P* < 0.01, and ^###^*P* < 0.001 by two-way repeated-measures ANOVA with LSD post hoc test. LVEDd: left ventricular end-diastolic diameter; LVEDs: left ventricular end-systolic diameter; LVPWd: left ventricular posterior wall thickness in end-diastole; LVPWs: left ventricular posterior wall thickness in end-systole; LVAWd: left ventricular anterior wall thickness in end-diastole; LVAWs: left ventricular anterior wall thickness in end-systole; EF: left ventricular ejection fraction; FS: left ventricular fractional shortening.

## Data Availability

The datasets used and analyzed during the current study are available from the corresponding author on reasonable request.

## Supplementary Materials

Supplementary Materials Primary and secondary antibodies used in this study were shown in Supplementary Table 1.
