# Supplementary material for: Protective Effect of Qiliqiangxin against Doxorubicin-Induced Cardiomyopathy by Suppressing Excessive Autophagy and Apoptosis
Source: Cardiovasc Ther. 2022 Jan 27;2022:9926635. doi: 10.1155/2022/9926635 (PMC8813302; doi:10.1155/2022/9926635)
Supplement: Supplementary Materials — Primary and secondary antibodies used in this study were shown in Supplementary Table 1. [file 9926635.f1.docx]

**Supplementary Table 1.** Primary and secondary antibodies used in this study

| Antibody | Customer | Product number | Dilution |
| --- | --- | --- | --- |
| LC3II/I | Cell Signaling Technology | 12741 | 1:1000 |
| BECN | ABclonal Technology | A7353 | 1:1000 |
| P62 | Cell Signaling Technology | 39749 | 1:1000 |
| Cleaved caspase 3 | Cell Signaling Technology | 9661 | 1:1000 |
| Cleaved caspase 9 | Cell Signaling Technology | 20750 | 1:1000 |
| Bax | Cell Signaling Technology | 2772 | 1:1000 |
| Bad | ABclonal Technology | A1593 | 1:1000 |
| Bcl2 | Cell Signaling Technology | 3498 | 1:1000 |
| BCL-xl | ABclonal Technology | A19703 | 1:1000 |
| ULK1 | ABclonal Technology | A8529 | 1:1000 |
| P-ULK1 | Cell Signaling Technology | 14202 | 1:1000 |
| m-TOR | ABclonal Technology | A11355 | 1:1000 |
| p-m-TOR | Cell Signaling Technology | 5536 | 1:1000 |
| PI3K | ABclonal Technology | A11402 | 1:1000 |
| p-PI3K | Cell Signaling Technology | 4228 | 1:1000 |
| AKT | ABclonal Technology | A18120 | 1:1000 |
| p-AKT | Cell Signaling Technology | 9271 | 1:1000 |
| GAPDH | ABclonal Technology | AC002 | 1:10000 |
| β-actin | ABclonal Technology | AC026 | 1:50000 |
| Peroxidase-conjugated AffiniPure Goat Anti-Rabbit IgG(H+L) | JacksonImmunoResearch | 111-035-003 | 1:5000 |
| Peroxidase-conjugated AffiniPure Goat Anti-Mouse IgG(H+L) | JacksonImmunoResearch | 115-005-003 | 1:5000 |
| Cy3 conjugated Goat Anti-Rabbit IgG(H+L) | Servicebio | GB21303 | 1:200 |
